# Was ist ausschlaggebend für die Gesamtqualität der postoperativen Schmerztherapie? Eine Frage der Perspektive

**DOI:** 10.1007/s00482-024-00839-5

**Published:** 2024-10-08

**Authors:** Paula Thomas, Thomas Weiss, Winfried Meissner, Philipp Baumbach

**Affiliations:** 1 Geschlechterforschung in der Medizin (GiM), Charité – Universitätsmedizin Berlin, Berlin, Deutschland; 2 Lehrstuhl für Klinische Psychologie, Friedrich-Schiller-Universität, Jena, Deutschland; 3 Klinik für Anästhesiologie und Intensivmedizin, Universitätsklinikum Jena, Friedrich-Schiller-Universität Jena, Jena, Deutschland; 4 Abteilung Palliativmedizin der Klinik für Innere Medizin II, Universitätsklinikum Jena, Friedrich-Schiller-Universität Jena, Jena, Deutschland

**Keywords:** Akutschmerz, Postoperativer Schmerz, Behandlungsqualität, „Patient-reported outcomes“, „Patient-reported outcome measures“, Domänen des Outcomes, Acute pain, Postoperative pain, Quality of care, Patient-reported outcomes, Patient-reported outcome measures, Outcome domains

## Abstract

**Hintergrund:**

Die Domänen *Schmerzintensität, schmerzbedingte Beeinträchtigung, Nebenwirkungen, Aufklärung, Partizipation* und *persönlicher Umgang* wurden bereits als relevant im Bereich perioperativer Schmerzen herausgestellt. Offen ist bisher, welche dieser Domänen besonders ausschlaggebend für die subjektiv empfundene Gesamtqualität der postoperativen Schmerztherapie sind.

**Ziel:**

In dieser Querschnittsstudie wurde mithilfe eines neu entwickelten Befragungsinstruments die Relevanz dieser Domänen für Patient*innen sowie vergleichend für Behandelnde erfasst.

**Methoden:**

Die Befragung der Patient*innen (*n* = 40) erfolgte am ersten postoperativen Tag am Universitätsklinikum Jena. Vergleichend wurden Angaben von 63 Behandelnden (Fachdisziplinen: *n* = 15 Anästhesiologie, *n* = 17 Chirurgie, *n* = 31 Pflege) erhoben. Das Befragungsinstrument umfasste primär alle paarweisen Vergleiche zwischen den Domänen im Hinblick auf die Gesamtqualität der postoperativen Schmerztherapie. Die daraus resultierenden Summenwerte für jede Domäne waren die primäre Zielgröße, die mittels *verallgemeinerter Schätzgleichungen *analysiert wurde.

**Ergebnisse:**

Innerhalb der Patient*innen zeigten sich signifikante Unterschiede in der Relevanz der Domänen, wobei *persönlicher Umgang *gefolgt von *Schmerzintensität *und *-beeinträchtigung *priorisiert wurde. Zudem ergaben sich innerhalb der Domänen signifikante Unterschiede zwischen Patient*innen und den befragten Fachdisziplinen sowie zwischen den Fachdisziplinen selbst.

**Schlussfolgerung:**

Patient*innen empfinden die Domänen *persönlicher Umgang* sowie Reduktion der *Schmerzintensität *und -*beeinträchtigung* entscheidend für die Gesamtqualität. Ob sich eine Harmonisierung der Sichtweisen von Patient*innen und Behandelnden positiv auf die Patient*innenzufriedenheit auswirkt, sollte weiter untersucht werden.

**Graphic abstract:**

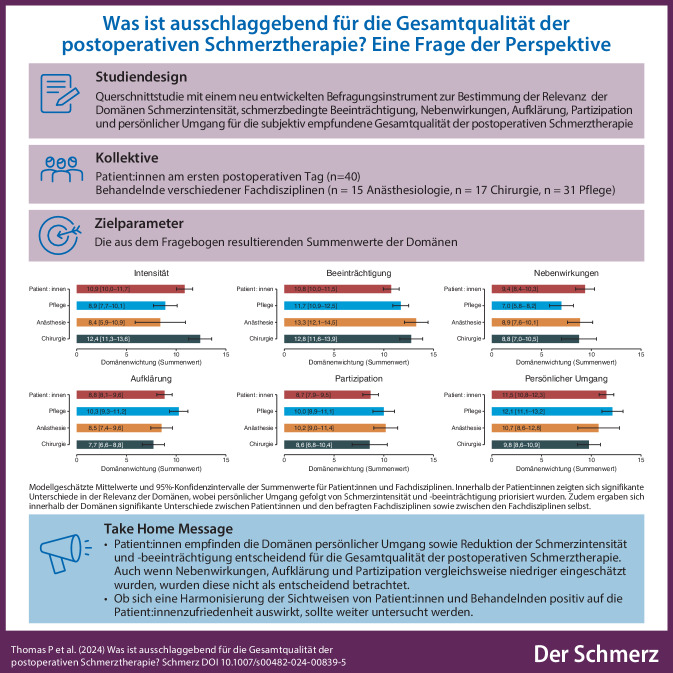

**Zusatzmaterial online:**

Die Online-Version dieses Beitrags (10.1007/s00482-024-00839-5) enthält ergänzende Informationen sowie die Befragungsinstrumente für Patient*innen und Behandelnde.

## Abstract (en)

**Background:**

The outcome domains *pain intensity*, *pain-related interference*, *side effects*, *(treatment) information*, *participation* and *personal interaction *have all been identified as relevant factors in the management of perioperative pain. However, it is not yet clear which of these are particularly significant for the subjectively perceived overall quality of postoperative pain management.

**Aim:**

A newly developed questionnaire was used in this cross-sectional study to assess the relevance of these domains for patients and compare the relevance to healthcare professionals (HCP).

**Methods:**

The patient survey (*n* = 40) was conducted on the first postoperative day at Jena University Hospital, Germany. In order to investigate group differences, 63 HCP (disciplines: *n* = 15 anaesthesiology, *n* = 17 surgery, *n* = 31 nursing) were recruited. The questionnaire primarily included all pairwise comparisons between the domains with regard to the overall quality of postoperative pain management. The resulting sum scores for each domain were the primary outcome measure, which were analysed using generalized estimating equations.

**Results:**

Within the group of patients, there were significant differences in the prioritization of the six outcome domains, with *personal interaction* followed by *intensity* and *interference* having received the highest ratings. There were also significant differences within the domains between the perspectives of patients and HCP, as well as between the HCP themselves.

**Conclusions:**

The study demonstrates that *personal interaction* and the reduction of *pain intensity* and *interference *are three key factors that are significant for patients’ experience of postoperative pain management. However, the extent to which the harmonisation of these three factors with those given prominence by HCP would positively impact postoperative pain management remains unclear and should be investigated further.

## Hintergrund und Fragestellung

Das übergeordnete Ziel der postoperativen Schmerztherapie ist neben dem physischen und psychischen Funktionszugewinn, das Leid der Patient*innen zu minimieren [14]. Die Abschätzung des Therapieerfolgs bei Schmerzen basiert neben objektivierbaren Variablen, wie etwa dem Analgetikabedarf, primär auf subjektiven Angaben von Patient*innen hinsichtlich ihres Gesundheitszustands (sog. „patient-reported outcomes“ [PRO]; [22]), deren standardisierte Erfassung mithilfe von „patient-reported outcome measures“, meist in Form validierter Fragebögen, erfolgt [10]. Neben der Schmerzintensität sollten zur Beurteilung des Therapieerfolgs, insbesondere im perioperativen Setting, weitere Facetten des Schmerzerlebens (sog. „outcome domains“ bzw. Ergebnisdomänen), wie etwa körperliche Funktionsfähigkeit oder Nebenwirkungen, herangezogen werden [11, 14].

In ähnlicher Weise wird in den deutschlandweiten bzw. internationalen Qualitätsverbesserungsprojekten QUIPS (Qualitätsverbesserung in der postoperativen Schmerztherapie; [13]) und PAIN OUT (Improvement in Postoperative Pain Outcome; [18]) mithilfe validierter Fragbögen, welche auf Vorarbeiten der *American Pain Society* basieren [9], die Gesamtqualität der perioperativen Schmerztherapie über mehrere Domänen erfasst. Für die vorliegende Untersuchung wurden gezielt die Domänen *Schmerzintensität* [9, 13, 14, 18]*, schmerzbedingte Beeinträchtigung* [9, 13, 14, 18], *Nebenwirkungen* [9, 13, 14, 18]*, Aufklärung* [9, 13, 18], *Partizipation* [9, 13, 18] und *persönlicher Umgang* betrachtet. Obgleich diese bereits als relevant herausgestellt wurden und Teil dieser langjährig zum Einsatz gebrachten Fragebögen sind, ist bisher offen, welche Domänen aus der Sicht von Patient*innen für die subjektiv empfundene Gesamtqualität der postoperativen Schmerztherapie ausschlaggebend sind. Damit leistet die vorliegende Untersuchung einen Beitrag zur „patient preference information“ (PPI), welche darauf abzielt, Präferenzen und Wünsche, im Sinne von positiven Outcomes und Sorge vor möglichen negativen Outcomes, und somit auch das Ausmaß der Akzeptanz bezüglich bestimmter Therapien patient*innenzentriert zu erfassen [2, 8].

Das primäre Ziel der vorliegenden Querschnittsstudie war es, aus der Perspektive der Patient*innen zu erfassen, welche der sechs Domänen für eine gute Gesamtqualität der postoperativen Schmerztherapie besonders relevant sind. Als sekundäres Ziel wurde ein Vergleich mit der Einschätzung von Behandelnden aus unterschiedlichen Fachdisziplinen angestrebt.

## Methodik

Die Arbeit entstand im Rahmen des multizentrischen, interdisziplinären Benchmark-Projekts QUIPS (DRKS00006153; [13]).

### Beschreibung der Domänen

Die betrachteten Domänen wurden primär auf Grundlage der in QUIPS und PAIN OUT verwendeten Fragebögen bzw. des revised American Pain Society Patient Outcome Questionnaire gewählt [9, 13, 18]. Hierbei wird das Konstrukt „Gesamtqualität der postoperativen Schmerztherapie“ aus der Perspektive von Patient*innen anhand einzelner Domänen festgemacht bzw. wird über diese definiert, was u. a. auch faktorenanalytisch gezeigt werden konnte [9, 18]. *Schmerzintensität *wurde mehrfach als relevante Domäne herausgestellt [9, 13, 14, 18]. Neben der Intensität wurde die *schmerzbedingte Beeinträchtigung*, z. B. hinsichtlich Bewegung, Schlaf oder Stimmung, als weitere relevante Domäne identifiziert [9, 13, 14, 18]. *Beeinträchtigung *erscheint mitunter relevant, da ein Therapieziel beinhaltet, selbstbestimmtes Handeln und somit eine Annäherung an die Funktionsfähigkeit vor der Operation zu erreichen oder sogar zu verbessern [11]. Die Reduktion oder Vermeidung von *Nebenwirkungen* (z. B. Übelkeit) ist ein weiteres wichtiges Therapieziel [9, 13, 14, 18]. Studienergebnisse zeigten bereits eine Assoziation zwischen dem Ausmaß der Nebenwirkungen und der Zufriedenheit mit der Schmerztherapie [21]. *Aufklärung* beinhaltet die Weitergabe von Informationen über Möglichkeiten der Schmerztherapie und *Partizipation* meint die Beteiligung an Entscheidungen zur Schmerztherapie [9, 13, 18]. Letztere wurde bereits als ein Korrelat für die Zufriedenheit mit dem Ergebnis der Schmerztherapie herausgestellt [21]. *Persönlicher Umgang* beschreibt die zwischenmenschliche Interaktion von Patient*innen und Behandelnden und wurde als zusätzliche Domäne mit aufgenommen. Sie lässt sich durch den Wunsch nach zugewandter Unterstützung, Berücksichtigung der eigenen Bedürfnisse und respektvollem Umgang skizzieren [7, 21].

### Studienteilnehmer*innen

Die Befragung aller Teilnehmenden fand zwischen Juni und November 2021 statt. Patient*innen wurden im Rahmen des QUIPS-Projekts am ersten postoperativen Tag auf Stationen des Universitätsklinikums Jena (UKJ) befragt. Die Behandelnden wurden intern am UKJ sowie extern via E‑Mail oder persönlichen Kontakt innerhalb des QUIPS-Netzwerks akquiriert (siehe auch Online-Zusatzmaterial A: 1 Ein- und Ausschlusskriterien).

Neben dem bereits bestehenden positiven Ethikvotum für die QUIPS-Studie (2722-12/09) wurde ein *Amendment* für die Zusatzbefragung eingeholt. Die schriftliche Einwilligung wurde von Patient*innen und Behandelnden eingeholt. Auf die Erhebung demografischer Variablen wurde bei den Behandelnden verzichtet.

### Befragungsinstrument

Das Befragungsinstrument basiert im Wesentlichen auf dem Verfahren der Prioritätenanalyse, an deren Ende eine individuelle Präferenzmatrix hinsichtlich der Domänen für jede*n Befragte*n vorliegt [4]. Im Detail wurden alle Domänen paarweise im Hinblick auf die Gesamtqualität der postoperativen Schmerztherapie verglichen. Die Formulierung für Patient*innen lautete: *„Welcher der einzelnen Bereiche erscheint aus Ihrer Sicht für die Gesamtqualität der Schmerztherapie nach Ihrer Operation jeweils wichtiger?“ *Für Behandelnde lautete die Formulierung: *„Welche der einzelnen Domänen erscheint aus Ihrer beruflichen Perspektive wichtiger für die Gesamtqualität der postoperativen Schmerztherapie?“*. Beiden Gruppen stand für jeden paarweisen Vergleich eine fünfstufige Bewertungsskala zur Verfügung (*„A deutlich wichtiger als B“, „A wichtiger als B“, „A und B gleich wichtig“, „A weniger wichtig als B“* und *„A deutlich weniger wichtig als B“*).

Aus den Bewertungen ergibt sich für jede Domäne ein Summenwert zwischen 0 und 20 (bei gleicher Gewichtung aller Domänen entsteht ein Summenwert von 10 für alle Domänen). Die Summenwerte der einzelnen Domänen bildeten die Hauptzielgröße der Studie.

Die Befragungsinstrumente für die Patient*innen und Behandelnden befinden sich im Online-Zusatzmaterial B und C. Der QUIPS-Ergebnis-Fragebogen ist im Online-Zusatzmaterial A: 2 QUIPS-Ergebnis-Fragebogen hinterlegt.

### Statistik

Allgemein wurden für kategoriale Variablen absolute und relative Häufigkeiten bestimmt. Für metrische Variablen wurde der Mittelwert (MW) und die Standardabweichung (SD) ermittelt.

Der primäre Endpunkt – Unterschiede in den Summenwerten der Domänen innerhalb der Patient*innen – wurde mithilfe verallgemeinerter Schätzgleichungen („generalized estimating equations“ [GEE]) analysiert (Arbeitskorrelationsmatrix: unstrukturiert, metrische abhängige Variable: Normalverteilung und Linkfunktion „Identität“; [23]). Entsprechend wurden die Summenwerte als abhängige Variable und die *Domäne* als 6‑stufige faktorielle unabhängige Variable modelliert. Die Modelleffekte der *Domäne* wurden mithilfe eines Wald-Tests analysiert. Für alle paarweisen Post-hoc-Vergleiche (Kontraste) der modellgeschätzten Randmittel, d. h. der vom Modell vorhergesagten Mittelwerte, wurde eine Adjustierung der *p*-Werte nach der Bonferroni-Holm-Methode vorgenommen.

Der sekundäre Endpunkt – Unterschiede in den Summenwerten der Domänen zwischen Patient*innen und Gruppen von Behandelnden – wurde ebenfalls mit einem GEE-Modell untersucht. Auch hier wurden die Summenwerte als abhängige Variable modelliert. Die *Domäne* (6-stufiger Faktor), *Gruppe* (4-stufiger Faktor: Patient*innen, Pflege, Anästhesie, Chirurgie) sowie die *Interaktion* aus beiden wurden als unabhängige Variablen modelliert. Die Effekte der Modellterme wurden mithilfe von Wald-Tests analysiert. Pro Domäne wurden alle paarweisen Post-hoc-Vergleiche (Kontraste) der modellgeschätzten Randmittel zwischen den Gruppen betrachtet. Da es sich dabei um die sekundäre Fragestellung handelte, wurde auf eine Adjustierung der *p*-Werte verzichtet.

Um ein Maß der Effektstärke zu erhalten, wurden beide Modelle zusätzlich mit den z‑standardisierten Summenwerten (MW ± SD: 0 ± 1) berechnet. Unterschiede in den modellgeschätzten Randmitteln können damit als Unterschiede in Standardabweichungen gewertet werden (Absolutwerte: > 0,2 kleiner, > 0,5 mittlerer und > 0,8 starker Effekt; [6]).

Alle Analysen wurden mit R (Version 4.1.0, Wien, Österreich) und R‑Studio (Version 1.4.1717, Boston, USA) durchgeführt [15, 19]. *P*-Werte < 0,05 wurden als statistisch signifikant betrachtet.

## Ergebnisse

**Abb. 1:**
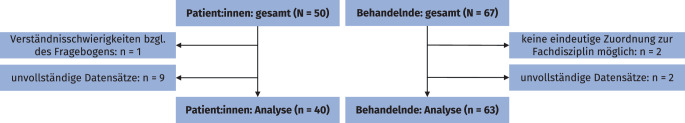
Übersicht zur Rekrutierung der Patient*innen und Behandelnden

Insgesamt standen vollständige Daten von 40 Patient*innen (*n* = 18/40 Frauen, 45 %; Alter, MW ± SD: 55,2 ± 16,8 Jahre) zur Verfügung (siehe Abb. 1; detaillierte Angaben im Online-Zusatzmaterial A: Tabelle E1). Die Patient*innen verteilten sich über verschiedene chirurgische Fachdisziplinen (Neurochirurgie: *n* = 22; Orthopädie/Traumatologie: *n* = 8; Frauenheilkunde/Geburtshilfe: *n* = 4; Allgemeinchirurgie: *n* = 2; Gefäßchirurgie: *n* = 2; Urologie: *n* = 1; Mund‑, Gesichts- und Kieferchirurgie: *n* = 1). Es standen vollständige Angaben von 63 Behandelnden (Pflege: *n* = 31; Anästhesie: *n* = 15; Chirurgie: *n* = 17) zur Verfügung.

### Perspektive der Patient*innen

**Abb. 2:**
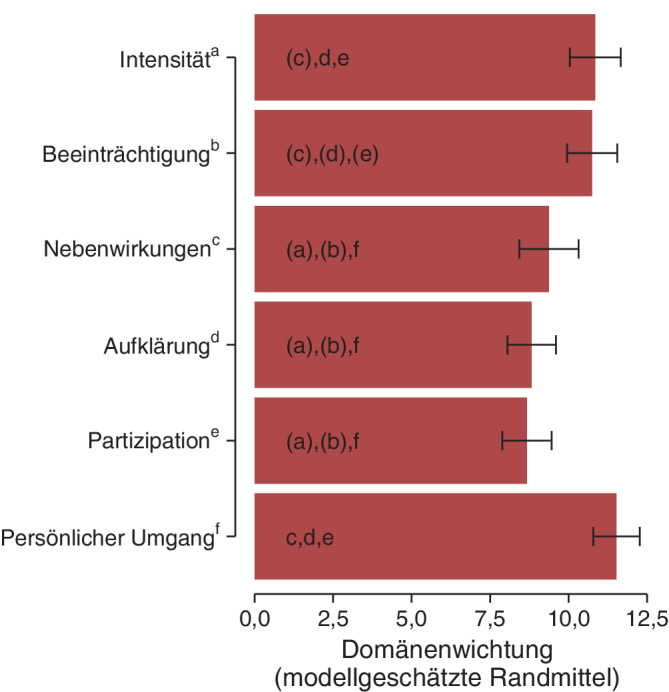
Modellgeschätzte Mittelwerte (sog. Randmittel, inklusive 95 %-Konfidenzintervalle) der Summenwerte der Domänen innerhalb der Gruppe der Patient*innen (*n* = 40). Die Buchstaben verweisen auf signifikante Unterschiede zwischen den Domänen (Bonferroni-Holm-korrigierte *p*-Werte < 0,05 der Post-hoc-Kontraste; Buchstaben in Klammern verweisen auf *p*-Werte < 0,05 vor der Bonferroni-Holm-Korrektur). So ergaben sich z. B. für Intensität (Index: a) signifikante Unterschiede zu Nebenwirkungen (Index: c), Aufklärung (Index: d) und Partizipation (Index: e), wobei der *p*-Wert für den Vergleich zwischen Intensität und Nebenwirkungen nach Bonferroni-Holm-Korrektur nicht mehr das Signifikanzniveau erreichte

Die deskriptiven Statistiken für die Summenwerte der Domänen innerhalb der Patient*innen sind in Tab. 1 zusammengefasst. Im Modell ergaben sich statistisch signifikante Unterschiede zwischen den Domänen (Modelleffekt *Domäne W*(5) = 26,2; *p* < 0,001). Die modellgeschätzten Randmittel sind in Tabelle E2 im Online-Zusatzmaterial A und in Abb. 2 zusammengefasst. Am wichtigsten schätzten die Patient*innen den *persönlichen Umgang* ein, direkt gefolgt von der *Schmerzintensität *und *Schmerzbeeinträchtigung*. Nach Korrektur auf multiples Testen wurde der *persönliche Umgang* signifikant relevanter eingeschätzt als *Nebenwirkungen, Aufklärung* und *Partizipation* (Abb. 2). Die *Schmerzintensität *wurde ebenfalls signifikant relevanter eingeschätzt als *Nebenwirkungen* und *Partizipation*. Die Effektstärken dieser Unterschiede bewegten sich im mittleren bis starken Bereich (Spannweite der Unterschiede in Standardabweichungen: 0,68–1,00).

**Tab. 1:** Deskriptive Darstellung der Summenwerte (0 bis 20) der Domänen für Patient*innen und Behandelnde (Pflege, Anästhesie und Chirurgie)

Domäne	Patient*innen (*n* = 40)	Behandelnde (*n* = 63)	Pflege (*n* = 31)	Anästhesie (*n* = 15)	Chirurgie (*n* = 17)
Intensität	10,8 (2,7)	9,7 (4,0)	8,9 (3,5)	8,4 (5,2)	12,4 (2,5)
Beeinträchtigung	10,8 (2,6)	12,4 (2,4)	11,7 (2,3)	13,3 (2,4)	12,8 (2,5)
Nebenwirkungen	9,4 (3,1)	7,9 (3,4)	7,0 (3,5)	8,9 (2,6)	8,8 (3,8)
Aufklärung	8,8 (2,5)	9,2 (2,7)	10,3 (2,7)	8,5 (2,2)	7,7 (2,4)
Partizipation	8,7 (2,6)	9,7 (3,2)	10 (3,1)	10,2 (2,5)	8,6 (3,8)
Persönlicher Umgang	11,5 (2,4)	11,2 (3,4)	12,1 (3,1)	10,7 (4,3)	9,8 (2,5)

Alle Ergebnisse in Mittelwert (Standardabweichung)

### Perspektive der Behandelnden

**Abb. 3:**
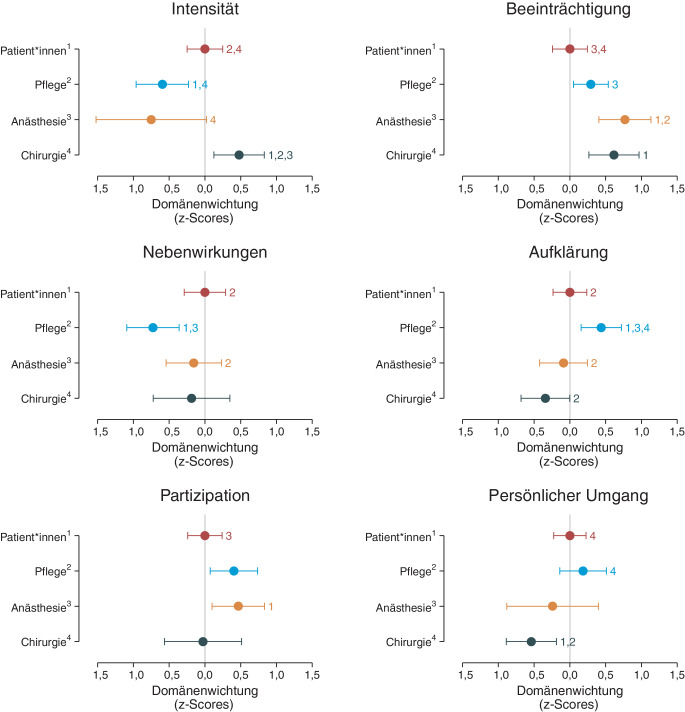
Grafische Darstellung der Modellergebnisse der z‑standardisierten Summenwerte der Domänen für die Gesamtstichprobe (Patient*innen und Behandelnde). Dargestellt sind die modellgeschätzten Randmittel (RM) inklusive 95 %-Konfidenzintervalle. Unterschiede zwischen den Gruppen (Post-hoc-Kontraste) sind entsprechend der z‑Standardisierung als Unterschiede in Standardabweichungen (Effektstärke) zu interpretieren. Um Unterschiede zu Patient*innen zu verdeutlichen, wurden die RM aller Gruppen zusätzlich auf die RM der Patient*innen zentriert, d. h., pro Domäne wurde von den RM der Gruppen stets das RM der Patient*innen abgezogen. Damit ergibt sich für Patient*innen stets ein Randmittel von 0 und positive bzw. negative Werte indizieren eine höhere bzw. niedrigere Wichtung der Domäne im Vergleich zu Patient*innen. Die farbigen Zahlen verweisen auf signifikante Unterschiede zwischen den Gruppen (1 = Patient*innen, 2 = Pflege, 3 = Anästhesie, 4 = Chirurgie). Es erfolgte keine Korrektur der *p*-Werte auf multiples Testen

Die deskriptiven Statistiken für die Summenwerte der Domänen für die Stichprobe der Patient*innen sowie Behandelnden sind in Tab. 1 zusammengefasst. Im Modell ergaben sich statistisch signifikante Unterschiede zwischen den Domänen (*W(5)* = 90,7; *p* < 0,001) und untersuchten Gruppen (*W*(3) = 24,9; *p* < 0,001; Interaktionsterm *Domäne* *×* *Gruppe W*(15) = 77,1; *p* < 0,001). Die modellgeschätzten Randmittel sind in Tabelle E3 im Online-Zusatzmaterial A und die Gruppenunterschiede in Abb. 3 zusammengefasst. Die Relevanz der Domäne *Schmerzintensität* schätzte die Chirurgie höher und die Pflege sowie Anästhesie niedriger ein als die Patient*innen. Im Vergleich zu den Patient*innen schätzten vor allem die Anästhesie und Chirurgie die Relevanz der *Schmerzbeeinträchtigung* höher ein. Die Relevanz von *Nebenwirkungen* wurde vor allem von der Pflege niedriger eingeschätzt als von den Patient*innen. Im Gegensatz dazu schätzte vor allem die Pflege die Relevanz von *Aufklärung* höher als die Patient*innen und anderen Behandelnden ein. Hinsichtlich der *Partizipation* schätzte die Anästhesie die Relevanz höher ein als die Patient*innen. In Bezug auf den *persönlichen Umgang* schätzte vor allem die Chirurgie die Relevanz geringer ein als Patient*innen und Pflege. Die Effektstärken aller signifikanten Unterschiede bewegten sich im geringen/mittleren bis starken Bereich (Spannweite der Unterschiede in Standardabweichungen: 0,44–1,23).

## Diskussion

In dieser Untersuchung wurde mithilfe eines neu entwickelten Befragungsinstruments die relative Relevanz der Domänen *Schmerzintensität, schmerzbedingte Beeinträchtigung, Nebenwirkungen, Aufklärung, Partizipation* und *persönlicher Umgang* hinsichtlich der Gesamtqualität der postoperativen Schmerztherapie ermittelt. Patient*innen betrachteten vor allem die Domäne *persönlicher Umgang* gefolgt von *Schmerzintensität* und -*beeinträchtigung* als relevant. Es ergaben sich signifikante Unterschiede zwischen Patient*innen und den befragten Fachdisziplinen sowie zwischen den Fachdisziplinen selbst.

### Perspektive der Patient*innen

Patient*innen schätzten die Domäne *persönlicher Umgang*, im Befragungsinstrument mit „Das Personal ist auf meine Wünsche bzgl. der Schmerztherapie eingegangen“ und „Ich habe den persönlichen Umgang als respektvoll erlebt“ beschrieben, am wichtigsten ein. Die Domäne spiegelt also ein globales Bedürfnis nach zwischenmenschlichem Kontakt, zugewandter Betreuung und guter Kommunikation zu den individuellen Behandlungszielen und Wünschen wider [7, 17]. Auch wenn diese Domäne nicht als klassische Ergebnisdomäne in Hinblick auf die Wirksamkeit der perioperativen Schmerzversorgung gewertet werden kann [14], scheint sie doch eine hohe Relevanz für die subjektive Gesamtbeurteilung der perioperativen Schmerztherapie zu haben. Inwieweit eine stärkere Zuwendung und ein wertschätzender Umgang mit den klassischen Outcomes assoziiert ist, muss weiter untersucht werden.

Neben dem *persönlichen Umgang* wurden *Schmerzintensität* und -*beeinträchtigung* als vorrangig betrachtet. Auch wenn zunehmend eine Betonung von *Aufklärung* und *Partizipation* im perioperativen Setting gefordert wird [8, 11, 14] und eine besondere Relevanz von *Partizipation* hinsichtlich der Patient*innenzufriedenheit in einer multinationalen Studie nachgewiesen werden konnte [21], deuten unsere Ergebnisse darauf hin, dass aus Patient*innensicht die *Schmerzintensität* und *-beeinträchtigung* von zentraler Bedeutung sind.

Es ist auch anzumerken, dass trotz der mittleren bis großen Effektstärken der Unterschiede innerhalb der Patient*innen die absoluten Unterschiede nicht sonderlich groß waren, d. h., alle untersuchten Domänen wurden auf Gruppenebene als bedeutsam erachtet.

### Perspektive der Behandelnden

*Schmerzintensität* wurde aus chirurgischer Sicht als relevanter eingeschätzt als aus Patient*innensicht, was auf einen professionsbedingten Fokus auf den Eingriff und die Schmerzintensität als eine direkte Folge zurückzuführen sein könnte [12]. Ein professionsbedingter Fokus bzw. ein Streben nach professioneller Selbstwirksamkeit lässt sich auch als mögliche Erklärung dafür anführen, dass *schmerzbedingte Beeinträchtigung *aus der Perspektive von Anästhesie und Chirurgie als relevanter eingeschätzt wurde als von Patient*innen. Es ist anzunehmen, dass die Wirksamkeit der durchgeführten und als adäquat wahrgenommenen Behandlung, so z. B. eine Reduzierung von Funktionseinschränkungen durch die Gabe von Medikation, einen wichtigen Aspekt der selbsteingeschätzten Behandlungsqualität ausmacht und eine wirksame Behandlung dabei mit Selbstwirksamkeit einhergeht [20]. Die Domäne *persönlicher Umgang* wurde aus der Perspektive von Pflegekräften und Patient*innen als relevanter eingeschätzt als von der Chirurgie. Der Pflege kommt durch den direkten Austausch mit den Patient*innen zu deren Wohlbefinden oftmals eine bestärkende Rolle zu [12]. Schmerz ist eine private Erfahrung [5, 16]. Die Pflege erhält durch den unmittelbaren Patient*innenkontakt womöglich einen gezielteren Einblick in das Schmerzerleben der Patient*innen [12].

Die Effektstärken der Gruppenvergleiche lagen im mittleren bis hohen Bereich, obgleich die absolute Höhe der Unterschiede auch hier weniger deutlich ausgeprägt war. D. h., auch von den befragten Fachdisziplinen wurden alle Domänen auf Gruppenebene als bedeutsam erachtet, wenn auch unterschiedlich nuanciert.

Abschließend ist festzuhalten, dass die Einschätzung der Patient*innen maßgeblich durch das subjektive Behandlungserleben geprägt ist und Präferenzen und Wünsche durch die unangenehme Qualität der Schmerzerfahrung motiviert sind [1, 3]. Die Fachdisziplinen hingegen scheinen sich, jeweils mit dem professionsbedingten Schwerpunkt, auf die Wirksamkeit der Behandlung zu fokussieren und die Relevanz der Domänen hinsichtlich der Umsetzung der Therapieziele zu bewerten, d. h., Leid möglichst zu minimieren sowie die physische und psychische Genesung zu unterstützen [11].

### Stärken, Limitationen und Ausblick

Stärken der Untersuchung sind der gezielte, quantitative Vergleich der Domänen mithilfe eines neu entwickelten Befragungsinstruments sowie der Einbezug der Perspektive von Patient*innen und verschiedenen Fachdisziplinen.

Zu den Limitationen zählen die monozentrische Befragung der Patient*innen, die relativ geringe Stichprobengröße aller befragten Gruppen sowie die nicht erhobenen soziodemografischen Informationen der Behandelnden. Die Auswahl der untersuchten Domänen erfolgte basierend auf Literaturrecherche und zum Großteil auf den in QUIPS bzw. PAIN OUT verwendeten Fragebögen. Die Ausweitung auf weitere Domänen sollte künftig zumindest erwogen werden. Es bleibt zu vermuten, dass der Befragungszeitpunkt der Patient*innen einen Einfluss auf die Einschätzungen genommen hat bzw. Präferenzen sich im weiteren postoperativen Verlauf verändern, z. B. dass mit zunehmendem zeitlichem Abstand zur Operation *Schmerzintensität* und *-beeinträchtigung* deutlicher in den Fokus rücken [21].

Zukünftige Untersuchungen in größeren Stichproben könnten potenziell Subgruppen von Patient*innen hinsichtlich der Präferenzen einzelner Domänen identifizieren. Diese könnten in Abhängigkeit demografischer oder behandlungsassoziierter Variablen stehen. Auch eine Analyse länderspezifischer Unterschiede wäre hochgradig interessant. Zudem wäre die Betrachtung potenzieller Zusammenhänge von individuellen Präferenzen und *PROs* von hohem Interesse. Eine Harmonisierung der Perspektiven könnte die Zufriedenheit von Patient*innen dadurch steigern, dass ihre Präferenzen und Bedürfnisse in den Bemühungen der unterschiedlichen Fachdisziplinen aufgegriffen werden.

### Schlussfolgerung

Aus Sicht der Patient*innen scheinen vor allem der *persönliche Umgang*, die *Schmerzintensität* und *-beeinträchtigung* relevant für die Gesamtqualität der postoperativen Schmerztherapie zu sein. Festgehalten werden muss, dass keine der untersuchten Domänen als gänzlich unwichtig eingeschätzt wurde. Auf Grundlage der Einschätzung der Domänen bezüglich ihrer Relevanz für die Gesamtqualität der postoperativen Schmerztherapie könnte in Zukunft ein gewichteter Qualitätsindex formuliert werden bzw. könnten sich konkrete Handlungsempfehlungen ableiten. Dafür ist die Erweiterung der Befragung auf eine größere und diversere Stichprobe notwendig.

## Fazit für die Praxis

- Aus Sicht der Patient*innen scheint vor allem der *persönliche Umgang* gefolgt von der *Schmerzintensität* und -*beeinträchtigung* relevant für die Gesamtqualität der postoperativen Schmerztherapie zu sein. Auch wenn die Domänen *Nebenwirkungen, Aufklärung* und *Partizipation* vergleichsweise niedriger eingeschätzt wurden, wurden diese nicht als irrelevant betrachtet.
- Zwischen Patient*innen und Behandelnden sowie zwischen Behandelnden einzelner Fachdisziplinen ergaben sich signifikante Unterschiede hinsichtlich der Relevanz der einzelnen Domänen.
- Für die Erfassung individueller Präferenzen steht das für diese Studie entwickelte Befragungsinstrument zur Verfügung.
- Inwieweit sich eine Harmonisierung der (individuellen) Sichtweisen von Patient*innen und Behandelnden positiv auf die Patient*innenzufriedenheit auswirkt, sollte Gegenstand weiterer Forschung sein.

## Supplementary Information

Online-Zusatzmaterial A_Ergänzende Informationen

Online-Zusatzmaterial B_Befragungsinstrument_Patient*innen

Online-Zusatzmaterial C_Befragungsinstrument_Behandelnde

## Data Availability

Die Daten, die die Ergebnisse dieser Publikation stützen, sind auf begründete Anfrage beim korrespondierenden Autor erhältlich.
