# Supplementary material for: What determines the overall quality of postoperative pain management? A question of perspective
Source: Schmerz. 2024 Oct 8;40(1):37–45. [Article in German] doi: 10.1007/s00482-024-00839-5 (PMC12858459; doi:10.1007/s00482-024-00839-5)
Supplement: Supplementary file 1 — Online-Zusatzmaterial A_Ergänzende Informationen [file 482_2024_839_MOESM1_ESM.pdf]

Online Zusatzmaterial: Ergänzende Informationen

1 Ein- und Ausschlusskriterien

1.1 Patient*innen

Einschlusskriterien:

- Mindestalter 18 Jahre
- schriftliche Einwilligungserklärung
- postoperativer Tag 1
- ausreichende Deutschkenntnisse

Ausschlusskriterien:

- Patient ist nicht in der Lage, den Fragebogen auszufüllen (sediert, verwirrt, fest schlafend)

1.2 Behandelnde

Einschlusskriterien:

- Mindestalter 18 Jahre
- schriftliche Einwilligungserklärung
- ausreichende Deutschkenntnisse
- Zuordnung in eine der Berufsgruppen: Anästhesie, Chirurgie oder Pflege

Es wurde die Befragung von 50 Patient*innen beantragt. Die geplante Fallzahl beruhte primär auf Machbarkeitsüberlegungen am Standort Jena und auf einer generischen Fallzahlabschätzung. Unter Anwendung eines gepaarten t-Tests bei einem zweiseitigen Signifikanzniveau von α = 5% lassen sich bei dieser Stichprobengröße (n = 50) kleine bis mittlere Effektstärken (Cohen's d ≥ 0,4) mit einer statistischen Power ≥ 80% detektieren. Zudem wurde die Befragung von bis zu 100 Behandelnden (mindestens n = 50) beantragt. Dies beruhte primär auf Machbarkeitsüberlegungen und einer generischen Fallzahlabschätzung. Unter Anwendung eines Zweistichproben t-Tests bei einem zweiseitigen Signifikanzniveau von α = 5% lassen sich ab einer Stichprobengröße (n = 50 pro Gruppe) mittlere Effektstärken (Cohen's d ≥ 0,57) mit einer statistischen Power ≥ 80% detektieren. Für die Fallzahlplanung wurde die *pwr.t.test* Funktion des R (Version 4.1.0) Pakets *pwr* (Version 1.3-0) genutzt.

2 QUIPS Ergebnis-Fragebogen

QUIPS Ergebnis-Fragebogen

Sehr geehrte Patientin, sehr geehrter Patient,

nachdem Sie sich zur Teilnahme entschlossen haben, bitten wir Sie nun den Fragebogen auszufüllen. Vielen Dank.

Bei den folgenden Fragen geht es um Ihre **Schmerzen seit Ihrer Operation.**

1. Wie stark waren Ihre Schmerzen seit der Operation, wenn Sie sich belastet haben, zum Beispiel bei **Mobilisierung, Bewegen, Waschen, Husten, Durchatmen?** Kreuzen Sie bitte die Zahl auf der Skala an, die für Ihren Belastungsschmerz zutrifft („0" bedeutet Schmerzfreiheit und „10" stärkster vorstellbarer Schmerz).

0	1	2	3	4	5	6	7	8	9	10

Kein Schmerz — Stärkster vorstellbarer Schmerz

2. Bitte kreuzen Sie hier den **stärksten Schmerz** an, den Sie seit Ihrer Operation hatten:

0	1	2	3	4	5	6	7	8	9	10

Kein Schmerz — Stärkster vorstellbarer Schmerz

3. Bitte kreuzen Sie hier den **geringsten Schmerz** an, den Sie seit Ihrer Operation hatten:

0	1	2	3	4	5	6	7	8	9	10

Kein Schmerz — Stärkster vorstellbarer Schmerz

Die nächsten vier Fragen beziehen sich darauf, ob bestimmte Tätigkeiten oder Ihre Stimmung seit der Operation **durch den Schmerz beeinträchtigt** sind.
Mit „beeinträchtigt" ist gemeint: die Tätigkeit ist **unmöglich** oder **nur unter großer Mühe möglich**.

Sind Sie durch die Schmerzen beeinträchtigt:

4. bei **Bewegung?**
❑ Ja ❑ Nein

5. beim **Husten oder tiefen Luftholen?**
❑ Ja ❑ Nein

6. beim **Schlafen?**
❑ Ja ❑ Nein

7. in Ihrer **Stimmung?**
❑ Ja ❑ Nein

8. Haben Sie sich seit der Operation sehr **müde** gefühlt?
❑ Ja ❑ Nein

9. Haben Sie seit der Operation unter **Übelkeit** gelitten?
❑ Ja ❑ Nein

10. Haben Sie seit der Operation unter **Schwindel** gelitten?
❑ Ja ❑ Nein

Copyright Winfried Meißner/ Uniklinikum Jena. Nutzung durch Dritte vorbehaltlich Genehmigung durch DGAI/ DGCH Version 3.2

11. Wurden Sie über die verschiedenen **Möglichkeiten Ihrer Schmerztherapie** informiert?

❑ Ja ❑ Nein

12. Wurden Sie in dem von Ihnen gewünschten Maß an **Entscheidungen** zu Ihrer **Schmerztherapie beteiligt**?

0	1	2	3	4	5	6	7	8	9	10

Gar nicht — Völlig ausreichend

13. Hätten Sie sich **MEHR Schmerztherapie** gewünscht, als Sie erhalten haben?

❑ Ja ❑ Nein

14. Bitte kreuzen Sie an, wie **zufrieden** Sie mit dem Ergebnis Ihrer **Schmerztherapie** seit Ihrer Operation sind:

0	1	2	3	4	5	6	7	8	9	10

Völlig unzufrieden — Sehr zufrieden

15. Haben Sie **nicht-medikamentöse** Methoden zur **Schmerzlinderung** benutzt oder erhalten?

❑ Ja ❑ Nein

Falls ja, welche?

❑ Kühlkompressen	❑ Wärme	❑ Meditation	❑ Tiefes Atmen
❑ Akupunktur	❑ Beten	❑ Umhergehen	❑ Massage
❑ Ablenkung (z. B. fernsehen, lesen)	❑ Vorstellungsbilder	❑ Entspannung	❑ TENS (Transkutane elektrische Nervenstimulation)
❑ Gespräche mit medizinischem Personal	❑ Gespräche mit Freunden/ Verwandten		

16. Hatten Sie **ständige Schmerzen**, die **drei Monate oder länger** andauerten, bevor Sie wegen dieser Operation ins Krankenhaus gekommen sind?

❑ Ja ❑ Nein

a. Falls ja, **wie stark** waren diese Schmerzen meistens?
Bitte kreuzen Sie den Wert an, der Ihre Schmerzintensität am besten widerspiegelt.

0	1	2	3	4	5	6	7	8	9	10

Kein Schmerz — Stärkster vorstellbarer Schmerz

b. Falls ja, **wo** traten diese **ständigen Schmerzen** auf?

❑ an der Körperstelle, die operiert wurde
❑ an einer anderen Körperstelle
❑ beides (Operationsstelle und woanders)

Vom Befragenden auszufüllen: Patient wurde interviewt: ❑ Ja ❑ Nein

Copyright Winfried Meißner/ Uniklinikum Jena. Nutzung durch Dritte vorbehaltlich Genehmigung durch DGAI/ DGCH Version 3.2

3 Ergänzende Tabellen

Tabelle E-1. Detaillierte Stichprobencharakterisierung basierend auf den Angaben der Patient*innen im QUIPS Fragebogen.

**Variablen**	**N=40**
**Demographie und Prozessvariablen**	
Alter (Jahre)	55,2 (16,8), n=40
Geschlecht (weiblich)	n=18/40 (45%)
ASA Klassifikation	
1	n=4/37 (10,8%)
2	n=29/37 (78,4%)
3	n=4/37 (10,8%)
Chronischer vorbestehender Schmerz	n=16/40 (40,0%)
Intraoperative Anästhesie	
Allgemeinanästhesie (solo)	n=29/37 (78,4%)
Regionalanästhesie (solo)	n=2/37 (5,4%)
Kombination	n=6/37 (16,2%)
Regionalanästhesie auf Normalstation	n=4/39 (10,3%)
Stationäre Therapieanordnung	n=37/39 (94,9%)
Schmerzdokumentation	n=6/39 (15,4%)
Opioide: Aufwachraum	n=15/31 (48,4%)
Opioide: Normalstation	n=16/38 (42,1%)
Non-Opioide: Aufwachraum	n=6/31 (19,4%)
Non-Opioide: Normalstation	n=31/38 (81,6%)
**Outcomevariablen**	
**Schmerzintensität (0–10 NRS)**	
unter Belastung	4,9 (3,3), n=39
maximal	5,8 (3,1), n=40
minimal	2,6 (2,7), n=39
**Schmerzbeeinträchtigung (ja)**	
Bewegung	n=30/39 (76,9%)
Husten/tief Luftholen	n=12/39 (30,8%)
Schlaf	n=21/38 (55,3%)
Stimmung	n=11/38 (28,9%)
**Nebenwirkungen (ja)**	
Müdigkeit	n=27/39 (69,2%)
Übelkeit	n=10/40 (25,0%)
Schwindel	n=15/40 (37,5%)
Wunsch nach mehr Schmerztherapie	n=8/36 (22,2%)
Zufriedenheit mit Schmerztherapie (0-10 NRS)	7,3 (2,7), n=38

Alle Angaben in n (%) oder Mittelwert (Standardabweichung); ASA, American Society of Anaesthesiologists; NRS, numerische Rating Skala; QUIPS, Qualitätsverbesserung in der postoperativen Schmerztherapie

Tabelle E-2. Modellergebnisse der Summenwerte der Domänen in der Stichprobe der Patient*innen.

Domäne	RM [95% KI]	Kontrast p <0,05
**Intensität** [a]	10,9 [10,0-11,7]	*(c), d, e*
**Beeinträchtigung** [b]	10,8 [10,0-11,5]	*(c), (d), (e)*
**Nebenwirkungen** [c]	9,4 [8,4-10,3]	*(a), (b), f*
**Aufklärung** [d]	8,8 [8,1-9,6]	*(a), (b), f*
**Partizipation** [e]	8,7 [7,9-9,5]	*(a), (b), f*
**Persönlicher Umgang** [f]	11,5 [10,8-12,3]	*c, d, e*

Modellgeschätzte Randmittel (inklusive 95% Konfidenzintervalle; RM [95%KI]). Signifikante Kontraste (p<0,05 nach Bonferroni-Holm Korrektur) zwischen den Domänen (a: Intensität; b: Beeinträchtigung; c: Nebenwirkungen; d: Aufklärung; e: Partizipation; f: persönlicher Umgang) sind in der letzten Spalte dargestellt. Indizes in Klammern verweisen auf p<0,05 vor der Bonferroni-Holm Korrektur.

Tabelle E–3. Modellergebnisse der Summenwerte der Domänen für die Gesamtstichprobe (Patient*innen und Behandelnde).

	**RM [95%KI]**	**Kontrast p <0,05**
**Intensität**		
Patient*innen [1]	10,9 [10,0-11,7]	*2; 4*
Pflege [2]	8,9 [7,7-10,1]	*1; 4*
Anästhesie [3]	8,4 [5,9-10,9]	*4*
Chirurgie [4]	12,4 [11,3-13,6]	*1; 2; 3*
**Beeinträchtigung**		
Patient*innen [1]	10,8 [10,0-11,5]	*3; 4*
Pflege [2]	11,7 [10,9-12,5]	*3*
Anästhesie [3]	13,3 [12,1-14,5]	*1; 2*
Chirurgie [4]	12,8 [11,6-13,9]	*1*
**Nebenwirkungen**		
Patient*innen [1]	9,4 [8,4-10,3]	*2*
Pflege [2]	7,0 [5,8-8,2]	*1; 3*
Anästhesie [3]	8,9 [7,6-10,1]	*2*
Chirurgie [4]	8,8 [7,0-10,5]	
**Aufklärung**		
Patient*innen [1]	8,8 [8,1-9,6]	*2*
Pflege [2]	10,3 [9,3-11,2]	*1; 3; 4*
Anästhesie [3]	8,5 [7,4-9,6]	*2*
Chirurgie [4]	7,7 [6,6-8,8]	*2*
**Partizipation**		
Patient*innen [2]	8,7 [7,9-9,5]	*3*
Pflege [2]	10,0 [8,9-11,1]	
Anästhesie [3]	10,2 [9,0-11,4]	*1*
Chirurgie [4]	8,6 [6,8-10,4]	
**Persönlicher Umgang**		
Patient*innen [1]	11,5 [10,8-12,3]	4
Pflege [2]	12,1 [11,1-13,2]	4
Anästhesie [3]	10,7 [8,6-12,8]	
Chirurgie [4]	9,8 [8,6-10,9]	*1; 2*

*Modellgeschätzte Randmittel (inklusive 95% Konfidenzintervalle; RM [95%KI]). Signifikante Kontraste (p<0,05) zwischen den Gruppen (1: Patient*innen; 2: Pflege; 3: Anästhesie; 4: Chirurgie) sind in der letzten Spalte dargestellt. In diesen Analysen erfolgte keine Korrektur der p Werte auf multiples Testen.*
