# Supplementary material for: What determines the overall quality of postoperative pain management? A question of perspective
Source: Schmerz. 2024 Oct 8;40(1):37–45. [Article in German] doi: 10.1007/s00482-024-00839-5 (PMC12858459; doi:10.1007/s00482-024-00839-5)
Supplement: Supplementary file 2 — Online-Zusatzmaterial B_Befragungsinstrument_Patient*innen [file 482_2024_839_MOESM2_ESM.pdf]

QUIPS – Qualitätsverbesserung in der postoperativen Schmerztherapie

Sehr geehrte Patientin, sehr geehrter Patient,

Sie haben bereits einige Fragen bezüglich der **Schmerzen seit Ihrer Operation** beantwortet. Die meisten dieser Fragen lassen sich bestimmten Bereichen zuordnen (z. B. Fragen bezüglich der Schmerzstärke).

Im Folgenden finden Sie eine kurze Beschreibung der einzelnen Bereiche:

Schmerzstärke

- Die Stärke meiner Schmerzen seit der Operation bei Belastung, zum Beispiel bei Mobilisierung, Bewegen, Waschen, Husten, Durchatmen.

Beeinträchtigung durch Schmerz

- Die Beeinträchtigung durch die Schmerzen bei Bewegung, beim Husten oder tiefen Luftholen, beim Schlafen, in meiner Stimmung.

Nebenwirkungen

- Symptome wie Müdigkeit, Übelkeit oder Schwindel seit der Operation.

Informationen über die Möglichkeiten der Schmerztherapie

- Information und Aufklärung über die verschiedenen Möglichkeiten meiner Schmerztherapie.

Beteiligung an Entscheidungen zur Schmerztherapie

- Ausreichende Beteiligung an Entscheidungen zu meiner Schmerztherapie und Einbindung in Therapieoptionen.

Persönlicher Umgang

- Das Personal ist auf meine Wünsche bzgl. der Schmerztherapie eingegangen.
- Ich habe den persönlichen Umgang (Pflegekräfte und Ärzt:innen) als respektvoll erlebt.

QUIPS-ID: |__|__|__|__|__|__|__|__|__|__|

Wir sind daran interessiert, **welche dieser Bereiche für die Gesamtqualität der Schmerztherapie nach Ihrer Operation besonders wichtig sind und welche der Bereiche für Sie weniger wichtig erscheinen**. Dafür haben wir auf den nächsten Seiten eine Gegenüberstellung aller Bereiche vorgenommen und bitten Sie um Ihre Einschätzung.

Bei den folgenden Fragen geht es um Vergleiche der oben genannten Bereiche. Bitte kreuzen Sie für jeden der Vergleiche eine Bewertung an:

A >> B bedeutet: A ist deutlich wichtiger als B

A > B bedeutet: A ist wichtiger als B

A = B bedeutet: A und B sind gleich wichtig

A < B bedeutet: A ist weniger wichtig als B

A << B bedeutet: A ist deutlich weniger wichtig als B

Hier ein kurzes Beispiel:

**A**				**B**
**Obst**				**Gemüse**
○ A >> B	⊕ A > B	○ A = B	○ A < B	○ A << B

Dieser Person ist Obst (A) wichtiger als Gemüse (B).

Bitte führen Sie nun auf den folgenden Seiten die Vergleiche für die Bereiche durch und geben Sie bitte jeweils an, welche der Bereiche aus Ihrer Sicht für die Gesamtqualität der Schmerztherapie nach Ihrer Operation wichtiger erscheinen.

Welcher der einzelnen Bereiche erscheint aus Ihrer Sicht für die Gesamtqualität der Schmerztherapie nach Ihrer Operation jeweils wichtiger?

A				B
**Schmerzstärke**				**Beeinträchtigung durch Schmerz**
○ A >> B	○ A > B	○ A = B	○ A < B	○ A << B
**Beeinträchtigung durch Schmerz**				**Informationen über die Möglichkeiten der Schmerztherapie**
○ A >> B	○ A > B	○ A = B	○ A < B	○ A << B
**Nebenwirkungen**				**Schmerzstärke**
○ A >> B	○ A > B	○ A = B	○ A < B	○ A << B
**Informationen über die Möglichkeiten der Schmerztherapie**				**Nebenwirkungen**
○ A >> B	○ A > B	○ A = B	○ A < B	○ A << B
**Beteiligung an Entscheidungen zur Schmerztherapie**				**Schmerzstärke**
○ A >> B	○ A > B	○ A = B	○ A < B	○ A << B

Welcher der einzelnen Bereiche erscheint aus Ihrer Sicht für die Gesamtqualität der Schmerztherapie nach Ihrer Operation jeweils wichtiger?

A				B
**Persönlicher Umgang von Seiten des Personals und der Ärzt:innen**				**Nebenwirkungen**
○ A >> B	○ A > B	○ A = B	○ A < B	○ A << B
**Schmerzstärke**				**Informationen über die Möglichkeiten der Schmerztherapie**
○ A >> B	○ A > B	○ A = B	○ A < B	○ A << B
**Beeinträchtigung durch Schmerz**				**Persönlicher Umgang von Seiten des Personals und der Ärzt:innen**
○ A >> B	○ A > B	○ A = B	○ A < B	○ A << B
**Nebenwirkungen**				**Beeinträchtigung durch Schmerz**
○ A >> B	○ A > B	○ A = B	○ A < B	○ A << B
**Informationen über die Möglichkeiten der Schmerztherapie**				**Beteiligung an Entscheidungen zur Schmerztherapie**
○ A >> B	○ A > B	○ A = B	○ A < B	○ A << B
**Beteiligung an Entscheidungen zur Schmerztherapie**				**Beeinträchtigung durch Schmerz**
○ A >> B	○ A > B	○ A = B	○ A < B	○ A << B

Welcher der einzelnen Bereiche erscheint aus Ihrer Sicht für die Gesamtqualität der Schmerztherapie nach Ihrer Operation jeweils wichtiger?

A				B
**Persönlicher Umgang von Seiten des Personals und der Ärzt:innen**				**Informationen über die Möglichkeiten der Schmerztherapie**
○ A >> B	○ A > B	○ A = B	○ A < B	○ A << B
**Schmerzstärke**				**Persönlicher Umgang von Seiten des Personals und der Ärzt:innen**
○ A >> B	○ A > B	○ A = B	○ A < B	○ A << B
**Nebenwirkungen**				**Beteiligung an Entscheidungen zur Schmerztherapie**
○ A >> B	○ A > B	○ A = B	○ A < B	○ A << B
**Beteiligung an Entscheidungen zur Schmerztherapie**				**Persönlicher Umgang von Seiten des Personals und der Ärzt:innen**
○ A >> B	○ A > B	○ A = B	○ A < B	○ A << B

Bitte listen Sie weitere Aspekte auf, die aus Ihrer Sicht für die Gesamtqualität der Schmerztherapie nach Ihrer Operation ebenfalls wichtig sind.

Hätten Sie sich **MEHR Schmerztherapie** gewünscht, als Sie erhalten haben?
○ nein<br>○ ja
**Falls ja, geben Sie bitte die Gründe dafür an:**<br>(z. B. zu starke Schmerzen oder mangelnde Aufklärung über medikamentöse/nicht medikamentöse Verfahren)

Bitte kreuzen Sie an, wie **zufrieden** Sie mit dem Ergebnis Ihrer **Schmerztherapie** seit Ihrer Operation sind.

0	1	2	3	4	5	6	7	8	9	10

völlig unzufrieden — sehr zufrieden

Bitte kreuzen Sie an, wie wichtig der jeweilige Bereich für **Ihre Zufriedenheit mit dem Ergebnis Ihrer Schmerztherapie** ist.
„0“ bedeutet dieser Bereich ist überhaupt nicht wichtig für Ihre Zufriedenheit und „10“ bedeutet dieser Bereich ist äußerst wichtig für Ihre Zufriedenheit.

Eine möglichst geringe **Schmerzstärke** ist mir bezüglich meiner Zufriedenheit…

0	1	2	3	4	5	6	7	8	9	10

überhaupt nicht wichtig — äußerst wichtig

Eine möglichst geringe **Beeinträchtigung durch die Schmerzen** ist mir bezüglich meiner Zufriedenheit…

0	1	2	3	4	5	6	7	8	9	10

überhaupt nicht wichtig — äußerst wichtig

Möglichst geringe **Nebenwirkungen der Schmerztherapie** sind mir bezüglich meiner Zufriedenheit…

0	1	2	3	4	5	6	7	8	9	10

überhaupt nicht wichtig — äußerst wichtig

Möglichst viele **Informationen über die Möglichkeiten der Schmerztherapie** sind mir bezüglich meiner Zufriedenheit…

0	1	2	3	4	5	6	7	8	9	10

überhaupt nicht wichtig — äußerst wichtig

Eine möglichst starke **Beteiligung an Entscheidungen zur Schmerztherapie** ist mir bezüglich meiner Zufriedenheit…

0	1	2	3	4	5	6	7	8	9	10

überhaupt nicht wichtig — äußerst wichtig

Ein möglichst guter **persönlicher Umgang** seitens des Personals ist mir bezüglich meiner Zufriedenheit…

0	1	2	3	4	5	6	7	8	9	10

überhaupt nicht wichtig — äußerst wichtig

Was war ggf. noch wichtig für Ihre Zufriedenheit mit dem Ergebnis Ihrer Schmerztherapie:
____
____
____

Dieser Fragebogen wurde ausgefüllt:
o von dem/der Patient:in selbst<br>o mit Hilfe des medizinischen Personals

Wir bedanken uns herzlich für Ihre Teilnahme und wünschen Ihnen alles Gute!
