# Supplementary material for: What determines the overall quality of postoperative pain management? A question of perspective
Source: Schmerz. 2024 Oct 8;40(1):37–45. [Article in German] doi: 10.1007/s00482-024-00839-5 (PMC12858459; doi:10.1007/s00482-024-00839-5)
Supplement: Supplementary file 3 — Online-Zusatzmaterial C_Befragungsinstrument_Behandelnde [file 482_2024_839_MOESM3_ESM.pdf]

Sehr geehrte Kolleginnen, sehr geehrte Kollegen,

wir möchten Ihnen gerne einige Fragen zum **Behandlungssetting der postoperativen Schmerztherapie** stellen. Wir beschäftigen uns mit verschiedenen Domänen, die für die **Behandlungsqualität am ersten postoperativen Tag** wichtig sind.

Im Folgenden finden Sie eine kurze Beschreibung der einzelnen Domänen:

**Schmerzstärke**
• Die Stärke der Schmerzen seit der Operation bei Belastung, zum Beispiel bei Mobilisierung, Bewegen, Waschen, Husten, Durchatmen.
**Beeinträchtigung durch Schmerz**
• Die Beeinträchtigung durch die Schmerzen bei Bewegung, beim Husten oder tiefen Luftholen, beim Schlafen, in der Stimmung.
**Nebenwirkungen**
• Symptome wie Müdigkeit, Übelkeit oder Schwindel seit der Operation.
**Informationen über die Möglichkeiten der Schmerztherapie**
• Information und Aufklärung der Patient:innen über die verschiedenen Möglichkeiten der Schmerztherapie.
**Beteiligung an Entscheidungen zur Schmerztherapie**
• Ausreichende Beteiligung der Patient:innen an Entscheidungen zur Schmerztherapie und Einbindung der Patient:innen in Therapieoptionen.
**Persönlicher Umgang**
• Eingehen auf Patient:innen-Wünsche bzgl. der Schmerztherapie.<br>• Respektvoller Umgang mit den Patient:innen.

Wir sind daran interessiert, **welche dieser Domänen Sie aus Ihrer beruflichen Perspektive als besonders wichtig für die Gesamtqualität der post-operativen Schmerztherapie erachten und welche der Domänen Ihnen weniger wichtig erscheinen**. Dafür haben wir auf den nächsten Seiten eine Gegenüberstellung aller Domänen vorgenommen und bitten Sie um Ihre Einschätzung.

Bitte beachten Sie: Es geht bei den Fragen nicht darum, dass Sie sich in die Patient:innen hineinversetzen, vielmehr möchten wir **Ihre professionelle Sichtweise** erfahren.

Bei den folgenden Fragen geht es um Vergleiche der oben genannten Bereiche. Bitte kreuzen Sie für jeden der Vergleiche eine Bewertung an:

A >> B bedeutet: A ist deutlich wichtiger als B

A > B bedeutet: A ist wichtiger als B

A = B bedeutet: A und B sind gleich wichtig

A < B bedeutet: A ist weniger wichtig als B

A << B bedeutet: A ist deutlich weniger wichtig als B

Hier ein kurzes Beispiel:

**A**				**B**
**Obst**				**Gemüse**
o A >> B	⊕ A > B	o A = B	o A < B	o A << B

Dieser Person ist Obst (A) wichtiger als Gemüse (B).

Bitte führen Sie nun auf den folgenden Seiten die Vergleiche für die Domänen durch und geben Sie bitte jeweils an, welche der Domänen aus Ihrer beruflichen Perspektive wichtiger für die Gesamtqualität der post-operativen Schmerztherapie erscheinen.

Welche der einzelnen Domänen erscheint aus Ihrer beruflichen Perspektive wichtiger für die Gesamtqualität der post-operativen Schmerztherapie?

A				B
**Schmerzstärke**				**Beeinträchtigung durch Schmerz**
o A >> B	o A > B	o A = B	o A < B	o A << B
**Beeinträchtigung durch Schmerz**				**Informationen über die Möglichkeiten der Schmerztherapie**
o A >> B	o A > B	o A = B	o A < B	o A << B
**Nebenwirkungen**				**Schmerzstärke**
o A >> B	o A > B	o A = B	o A < B	o A << B
**Informationen über die Möglichkeiten der Schmerztherapie**				**Nebenwirkungen**
o A >> B	o A > B	o A = B	o A < B	o A << B
**Beteiligung an Entscheidungen zur Schmerztherapie**				**Schmerzstärke**
o A >> B	o A > B	o A = B	o A < B	o A << B

Welche der einzelnen Domänen erscheint aus Ihrer beruflichen Perspektive wichtiger für die Gesamtqualität der post-operativen Schmerztherapie?

A				B
**Persönlicher Umgang von Seiten des Personals und der Ärzt:innen**				**Nebenwirkungen**
o A >> B	o A > B	o A = B	o A < B	o A << B
**Schmerzstärke**				**Informationen über die Möglichkeiten der Schmerztherapie**
o A >> B	o A > B	o A = B	o A < B	o A << B
**Beeinträchtigung durch Schmerz**				**Persönlicher Umgang von Seiten des Personals und der Ärzt:innen**
o A >> B	o A > B	o A = B	o A < B	o A << B
**Nebenwirkungen**				**Beeinträchtigung durch Schmerz**
o A >> B	o A > B	o A = B	o A < B	o A << B
**Informationen über die Möglichkeiten der Schmerztherapie**				**Beteiligung an Entscheidungen zur Schmerztherapie**
o A >> B	o A > B	o A = B	o A < B	o A << B
**Beteiligung an Entscheidungen zur Schmerztherapie**				**Beeinträchtigung durch Schmerz**
o A >> B	o A > B	o A = B	o A < B	o A << B

Welche der einzelnen Domänen erscheint aus Ihrer beruflichen Perspektive wichtiger für die Gesamtqualität der post-operativen Schmerztherapie?

A				B
Persönlicher Umgang von Seiten des Personals und der Ärzt:innen				Informationen über die Möglichkeiten der Schmerztherapie
o A >> B	o A > B	o A = B	o A < B	o A << B
Schmerzstärke				Persönlicher Umgang von Seiten des Personals und der Ärzt:innen
o A >> B	o A > B	o A = B	o A < B	o A << B
Nebenwirkungen				Beteiligung an Entscheidungen zur Schmerztherapie
o A >> B	o A > B	o A = B	o A < B	o A << B
Beteiligung an Entscheidungen zur Schmerztherapie				Persönlicher Umgang von Seiten des Personals und der Ärzt:innen
o A >> B	o A > B	o A = B	o A < B	o A << B

Bitte listen Sie weitere Aspekte auf, die Ihnen für die Gesamtqualität der post-operativen Schmerztherapie ebenfalls wichtig erscheinen.

**Dieser Fragebogen wurde ausgefüllt:**
O von einem/einer Chirurg:in<br>O von einem/einer Anästhesist:in<br>O von einer Person des ärztlichen Stationspersonals<br>O von einer Person des Pflegepersonals

Wir bedanken uns herzlich für Ihre Teilnahme!
